# Type 2 Diabetes Mellitus-Induced Hyperglycemia in Patients with NAFLD and Normal LFTs: Relationship to Lipid Profile, Oxidative Stress and Pro-Inflammatory Cytokines

**DOI:** 10.3797/scipharm.1104-21

**Published:** 2011-05-29

**Authors:** Mohamed E. E. Shams, Mohammed M. H. Al-Gayyar, Enaase A. M. E. Barakat

**Affiliations:** 1 Department of Pharmaceutics, Faculty of Pharmacy, University of Mansoura, Mansoura, Egypt; 2 Department of Biochemistry, Faculty of Pharmacy, University of Mansoura, Mansoura, Egypt; 3 Program in Clinical and Experimental Therapeutics, College of Pharmacy, University of Georgia, USA; 4 Charlie Norwood Veterans Affairs Medical Center, Augusta, GA, USA; 5 Department of Internal Medicine, Faculty of Medicine, University of Mansoura, Mansoura, Egypt

**Keywords:** Nonalcoholic Fatty Liver Disease (NAFLD), Type 2 diabetes, Obesity, Lipid profile, Oxidative stress, Antioxidants, TNF-α, IL-6

## Abstract

Type 2 diabetes mellitus is associated with dyslipdemia, insulin resistance and non alcoholic fatty liver disease. The purpose of the current study was to assess whether type 2 diabetes mellitus-induced hyperglycemia has an effect on the lipid profile and release of oxidative stress markers and inflammatory mediators in patients with non alcoholic fatty liver disease and normal liver function tests which may in turn lead to enhancing the pathogenicity of this liver disease. For this purpose, one hundred and five outpatients, matched in age and weight, were classified into two groups: the first group consisted of patients with non alcoholic fatty liver disease and the second group consisted of patients with non alcoholic fatty liver disease in conjunction with hyperglycemia due to the presence of type 2 diabetes mellitus. In all patients, lipid profile, oxidative stress, and inflammatory mediators were assessed by measuring serum concentrations of triglycerides, low density lipoprotein, hydrogen preroxide, malondialdehyde, tumor necrosis factor-alpha and interleukin-6, respectively. In the studied population, it was found that the presence of type 2 diabetes mellitus-induced hyperglycemia significantly impaired lipid profile, and significantly enhanced the formation of hydrogen preroxide and malondialdehyde as well as significantly increased the release of tumor necrosis factor-alpha and interleukin-6 in the second group of patients. In addition, plasma glucose level showed significant positive correlation with hydrogen peroxide, malondialdehyde, tumor necrosis factor-alpha and interleukin-6. From the previous results, it was concluded that the presence of type 2 diabetes mellitus-induced hyperglycemia results in significant increase in lipid profile, oxidative stress markers and inflammatory mediators in patients with non alcoholic fatty liver disease and normal liver function tests. For this reason, further research studies may be essential to evaluate the benefit of adding suitable antioxidant and anti-inflammatory drugs to the treatment regimen for this group of patients. In addition, regular monitoring of blood glucose levels and liver function tests should be advised to this category of patients to reduce liver fat deposition and avoid the development of non alcoholic steatohepatitis, cirrhosis or liver cancer and their related complications.

## Introduction

Nonalcoholic Fatty Liver Disease (NAFLD) has become a global epidemic, affecting 20–40% of the general adult population [1]. During the past 20 to 30 years, the frequency of patients presenting with NAFLD has increased gradually in proportion to the increase in the population with life-style related diseases [2]. A recent study in Japan showed that approximately 20 to 25% of diabetic patients showed NAFLD [3]. Nonalcoholic fatty liver disease represents a spectrum of disorders characterized by predominantly macro-vesicular hepatic steatosis that occur in individuals even in the absence of consumption of alcohol in amounts considered harmful to the liver [1]. The likelihood of having NAFLD is directly proportional to body weight and presence of diabetes mellitus (DM) [4]. Most individuals who are suffering from NAFLD in its uncomplicated form are asymptomatic [5]. However, a subset progresses to more severe manifestations of the NAFLD disease spectrum may occur, including nonalcoholic steatohepatitis (NASH), fibrosis, cirrhosis and liver failure [6, 7].

It is estimated also that 75% of type 2 diabetic patients (T2DM) present some form of NAFLD of different degrees. It is ranging from simple fatty liver (steatosis), to nonalcoholic steatohepatitis (NASH), to cirrhosis (irreversible, advanced scarring of the liver). All of the stages of NAFLD have in common the accumulation of fat (fatty infiltration) in the liver cells (hepatocytes). In NASH, the fat accumulation is associated with varying degrees of inflammation (hepatitis) and scarring (fibrosis) of the liver [8].

Type 2 diabetes mellitus enhances lipolysis and inhibits glucose uptake thus increasing triglyceride (TG) formation by adipose tissue [9]. Adipose tissue has currently been regarded as an active player in the regulation of metabolism since the discovery of several adipocyte-derived factors, collectively known as adipokines. In this context, numerous substances, mainly released by adipose tissue such as tumor necrosis factor-alpha (TNF-α) are closely linked to each other and are thought to contribute to peripheral insulin resistance [10]. In addition, treatment of T2DM with insulin induces accumulation of lipids in the liver and skeletal muscle without affecting whole-body insulin sensitivity after near-normoglycemia for 67 h [11].

The main idea of the current research study was to examine the ability of T2DM-induced hyperglycemia in enhancing the pathogenicity of NAFLD by induction of oxidative stress (hydrogen peroxide and malondialdehyde) and releasing of inflammatory mediators (TNF-α and IL-6) in absence of liver damage as confirmed by the presence of normal liver function tests (LFTs). No previous studies measured this pathway in the early stages of the disease. Moreover, we aimed to provide a pharmaceutical care plan to chronically ill patients who are suffering from NAFLD in conjunction with T2DM.

## Materials and methods

### Patients’ characteristics

One hundred and five Patients were enrolled in the current study. They were selected from the outpatient clinics of Internal Medicine department, Specialized Medical Hospital, Mansoura University. Patients’ consent was obtained according to the regulations of the Egyptian Ministry of Health and the study design was approved by the local ethics committee.

The Inclusion criteria for all participants were:

- age between 40–60 years
- newly diagnosis of NAFLD and/or hyperglycemia or HbA1c level > 7 due to T2DM
- good general health condition other than T2DM or NAFLD.

The exclusion criteria were:

- history of alcohol ingestion
- malignancy
- previous gastrointestinal tract surgery
- smoking
- presence of any liver disease that can cause fatty liver such as chronic hepatitis C, autoimmune liver disease and Wilson’s disease
- ingestion of drugs known to produce hepatic steatosis such as corticosteroids, high-dose estrogens, methotrexate, valproic acid, tetracycline hydrochloride, amiodarone, isoniazide, phenytoin, carbamazepine or tamoxifen citrate in the previous 6 months
- high serum level of ALT, AST or gamma glutamyl transferase (GGT) (> 45 U/l).

### Diagnosis of NAFLD

Nonalcoholic Fatty Liver Disease was diagnosed by imaging tests, such as ultrasound, magnetic resonance imaging (MRI), computerized tomography scan (CT) and magnetic resonance elastography. In addition, blood tests were performed to assess liver function and to exclude other causes of liver disease. The exclusion of significant alcohol intake was essential. Presence of abnormal fat accumulation in the liver found by X-rays and ultrasound images confirmed the diagnosis. In very rare instances, a liver biopsy was performed.

### Diagnosis of T2DM

T2DM was diagnosed according to Health Care Guideline, 14th edition: http://www.icsi.org/diabetes_mellitus__type_2/management_of_type_2_diabetes_mellitus__9.html

Patients with fasting plasma glucose level ≥7.0 mmol/l and 2-h postload plasma glucose level ≥11.1 mmol/l on a 75-g oral glucose tolerance test were diagnosed as having type 2 diabetes. Because of the age range of the study population, all cases of diabetes were diagnosed after the age of 40 years and were thus classified as type 2 diabetes.

### Study design

Patients were classified into two groups, the first one (Group A) consists of patients suffering from NAFLD and the second group (Group B) consists of patients with NAFLD with normal liver function tests (LFTs) in conjunction with hyperglycemia due to T2DM. All prospective patients were subjected to patient history taking (personal, past and family history) as well as physical examinations. All subjects were interviewed for completion of a standardized questionnaire regarding personal medical history, current treatments, and life-style behaviors (see: http://www.signaturehealth.net/documents/OBGYNHP/...HistoryNew.pdf). Clinical parameters were also recorded including age, weight, height, hip and waist measurements and blood pressure. Body mass index (BMI) and waist/hip ratio (WHR) were also calculated. Safety parameters were assessed during clinic visit. Any patient who developed serious adverse effects, including heart or hepatic failure, or any episode of severe hypoglycemia, was excluded from the study.

### Assessment of medication adherence

Patients’ adherence to medication was assessed using the Measure Treatment Adherence (MTA) Scale developed by Delgado and Lima [12].

### Analysis of clinical laboratory and biochemical parameters

Venous blood samples were obtained from patients after overnight fasting (12 h). Serum ALT, AST and GGT were analyzed using commercially available kits. Serum TGs, Cholesterol and HDL-cholesterol were determined by commercially available kits from Human Company. Serum LDL-cholesterol was calculated by the equation of Friedewald [13].

The insulin resistance (HOMA IR) was calculated using the formula of Mathews [14]: HOMA IR = (fasting insulin X fasting plasma glucose) /22.5 (in case of glucose concentration mmol/l and 405 in case of glucose concentration mg/dl).

Leucocytic hydrogen peroxide concentration was measured by horseradish peroxidase (HRPO) method after modification as described previously by Al-Gayyar [15]. This method depends on the HRPO mediated oxidation of phenol red by H_2_O_2_ released from leucocytes, which resulted in the formation of a compound that could be read at 610nm.

Serum MDA was measured by thiobarbituric acid method using Al-Gayyar’s modifications [15]. In brief, serum proteins are precipitated by the addition of trichloroacetic acid. Then, thiobarbituric acid reacts with MDA to form thiobarbituric acid-reactive substance that is measured at 532 nm. Serum TNF-α level was analyzed using commercially available ELISA kits from BioSource International Inc, Calif, USA. Serum IL-6 was estimated by Pelikine Compact Human IL6 ELISA Kit

### Data management and statistical analysis

Mean values ± standard error (SE) was used for statistical computations using the computer software GraphPad InStat version 3.00, GraphPad Software, San Diego California USA. For group comparison ANOVA and Tukey-Kramer Multiple Comparisons Test was calculated. Statistical significance was predefined as P ≤0.05.

## Results and Discussion

Clinical characteristics of the patients’ population were summarized in table 1. Patients in group A and group B showed comparable age, blood pressure, body mass index and liver enzymes. The only difference which was found is that patients in group B had significant higher fasting blood glucose levels and insulin resistance compared with patients in group A.

### T2DM induced hyperglycemia alters lipid profile in NAFLD patients with normal LFTs

As shown in table 2, patients in group B showed significant increase in serum levels of triglycerides and LDL-C compared with those in group A (p<0.05). Patients in group A and group B showed no significant differences in their serum cholesterol level. In addition, patients in group B showed significant decrease in serum HDL-C concentration as compared with patients in group A (p<0.05).

T2DM is commonly associated with dyslipidemia and hyperglycemia in NAFLD patients [7, 16]. Therefore, our study showed significant impaired lipid profile in group B of the patients compared with patients in group A. Similar findings were found also by other researchers [7, 17]. Of note, the increased in TG in the patients was associated with insulin resistance. However, insulin resistance leads to hepatocytes fat deposition by two pathways; lipolysis and hyperinsulinemia. The first mechanism involves the suppression of the sensitivity of insulin receptor on lipocyte membrane leading increased free fatty acid amount more than the body needs which results in lipid deposition in hepatocytes. In the second mechanism, hyperinsulinemia blocks mitochondrial fatty-acid oxidation, resulting in a high concentration of intracellular fatty acids leading to oxidative stress [18].

### T2DM induced hyperglycemia potentiates release of oxidative stress in NAFLD patients with normal LFTs

**Fig. 1.**
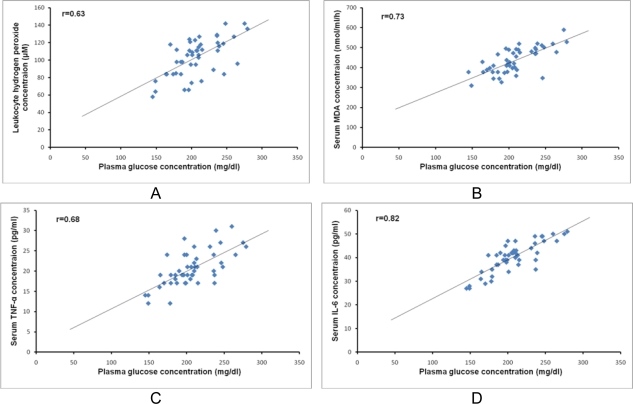
Significant positive correlations between plasma glucose level and the following parameters: A) leukocyte hydrogen peroxide concentration (r=0.63 and p<0.01), B) serum malondialdehyde (MDA) concentration (r=0.73 and p<0.01), C) serum tumor necrosis factor alpha (TNF-α) (r=0.68 and p<0.01) and D) serum IL-6 concentration (r=0.82 and p<0.01.)

Patients in group B showed a significant increase in leukocyte level of hydrogen peroxide and serum concentration of malondialdehyde (MDA) compared with those in group A, p<0.05 (Table 2). In addition, as shown in figure 1 (A and B), there were significant positive correlations between plasma glucose level and both leukocyte hydrogen peroxide concentration (r=0.63 and p<0.01) and serum MDA concentration (r=0.73 and p<0.01).

Interestingly, a significant increase in leukocyte level of hydrogen peroxide was found in patients of group B compared with patients in group A. There were no previous studies that measured the hydrogen peroxide levels in patients with both NAFLD and T2DM. However, some previous studies reported an increase in oxidative stress in patients with NAFLD and/or T2DM [19–22]. The increase in the oxidative stress can be explained by hyperglycemia which can induce oxidative stress by several mechanisms such as glucose auto-oxidation, increased nitric oxide synthase activity and FFAs oxidation [19]. The accumulated hydrogen peroxide could undergo the Fenton’s reaction, generating hydroxyl radical, which may lead to lipid peroxidation. The plasma and mitochondrial membrane, consists primarily of lipids, are vulnerable to free radical damage and ROS-induced lipid peroxidation leading to cell dysfunction and adipose tissue damage resulting in the formation of lipid degradation products such as MDA.

### T2DM induced hyperglycemia enhances the release of proinflammatory cytokines in NAFLD patients with normal LFTs

As shown in table 2, patients in group B showed significant increase in serum concentrations of TNF-α and IL-6 as compared with patients in group A (p<0.05). Moreover, significant positive correlations were observed between plasma glucose concentration and both serum TNF-α (r=0.68 and p<0.01, Figure 1 (C) and serum IL-6 concentration (r=0.82 and p<0.01, Figure 1 (D).

Interestingly, we found a significant increase in serum level of MDA in patients of group B compared with those in group A, which is in agreement with the findings in other research studies [23, 24]. The produced MDA is a reactive compound, which causes a toxic stress in cells and stimulates the release of inflammatory mediators such as TNF-α and IL-6. Therefore, we found a significant increase in both markers in patients of group B compared with patients in group A. However, no previous studies investigated the levels of inflammatory mediators in patients with both T2DM and NAFLD. The increased expressions of TNF-α and its receptor have been previously reported in liver and adipose tissue in obese individuals with NASH, which correlates with the degree of fibrosis [25]. The increased production of inflammatory cytokines in NAFLD could exacerbate systemic and hepatic insulin resistance with worsening inflammation and fibrosis. Inflammatory cytokines cause impaired insulin signalling, cell damage, neutrophil chemotaxis, hepatic stellate cell activation and apoptosis.

In fact, there is an interactive relationship between oxidative stress markers and inflammatory mediators. Increases in TNF expression induce activation of NAD(P)H oxidase and production of reactive oxidative species, leading to endothelial dysfunction in type 2 diabetes [26]. On the other hand, oxidative stress has also been shown to increase production of pro-inflammatory cytokines (TNF-α, TGF-α, IL-6, IL-8) [27, 28].

It has recently been hypothesized that NAFLD is a consequence of a ‘two hit’ insult. As a consequence of insulin resistance, there is excessive accumulation of triglyceride in the hepatocytes, this being the first hit. While, oxidative stress and increased inflammatory cytokines were considered as the second hit, resulting in hepatocyte injury, inflammation and fibrosis [29].

### Patients’ adherence to anti-diabetic medication

The patients involved in the current study were newly diagnosed with NAFLD either with or without hyperglycemia due to T2DM. Patients in group B demonstrated unsatisfactory adherence to anti-diabetic medication therapy, dietary/exercise recommendations and even appointments to show up at the clinics for the scheduled check up. High fasting blood glucose levels [(mean± SE) equal 203.6±4.7 mg/dl)] were found in this group as shown in table 1. All of these patients required pharmacological treatment to control their blood glucose levels. Of all patients in this group [N= 47(100%)], the majority [N=32 (68.0%)] treated with basal insulin [bedtime intermediate-acting insulin (NPH, Lente or Mixtard 30/70) or bedtime long-acting insulin (Glargine)] as a monotherapy (N=7) or in combination with Metformin (N=16) or with Gliclazide MR (N=9). On the other hand, two patients (4.2%) treated with Metformin monotherapy, seven patients treated with Gliclazide MR monotherapy (14.9%), four patients (8.5%) treated with a combination therapy of Metformin and Gliclazide MR, while two patients only treated with Metformin and Pioglitazone (4.2%).

The majority of patients in group B either treated with insulin monotherapy or with a combination therapy of insulin with an oral hypoglycemic agent (OHA) because these patients were inadequately controlled with monotherapy of OHA. Despite the pharmacological treatment in this group of patients, hyperglycemia was found. This may be due to irregular monitoring of blood glucose level and/or due to unsatisfactory adherence to medication therapy, dietary/exercise and even appointment to show up at the clinics for the scheduled check up. An improvement with medication adherence may be achieved through continuing patient education about the disease encouraging patients to monitor their blood glucose level regularly [30].

Two goals are present as a strategy in treatment of patients who are suffering from NAFLD in conjunction with T2DM to improve the quality of life and prolong the survival. The primary target is to encouraging them to change their lifestyles and strength self-monitoring through patient education by clinicians. This includes: ingestion of a healthy food containing a low carbohydrate and low-fat balanced diet, a reduction of drinks containing sugar, saturated fat and trans-fat intake, an increase in dietary fiber content, taking moderate aerobic exercise at least 4 times per week, moderate weight reduction. In addition regular measuring and monitoring of blood glucose levels and LFTs at which glycated hemoglobin (HbA1C) should ideally be brought to < 7 [31]. The secondary target is to reduce liver fat deposition and avoid the development NASH, cirrhosis or liver cancer and their related complications. Liver protective and anti-inflammatory drugs may be beneficial drugs in these patients to prevent and treat steatohepatitis and advanced fibrosis [32]. One to two kinds of liver protective, antioxidant and anti-inflammatory drugs such as polyene phosphatidylcholine, silymarin, glycyrrhizin, bicyclol, vitamin E, ursode-oxycholic acid and S-ademetionine, insulin sensetizer could be used, according to the disease activity, the stage of the disease, drug efficacy and the cost of treatment [33].

## Conclusion

From the previous results, we can conclude that:

1. T2DM induced hyperglycemia may enhance the pathogenicity of NAFLD by increasing triglycerides, LDL-C and glucose formation.
2. Hyperglycemia in T2DM enhances the formation of oxidative stress markers and inflammatory mediators triggering cell dysfunction.
3. The release of inflammatory mediators in patients who are suffering from both T2DM and NAFLD occurs in an early stage of the disease even before liver damage. Diabetes educators and clinicians should emphasize the benefits of a healthy lifestyle, regular monitoring of blood glucose levels, HbA1c and lipid profile especially in patients who are suffered from NAFLD to protect them against a progressive liver damage.

## Figures and Tables

**Tab. 1.** Clinical characteristics of patients with NAFLD in comparison to the patients with NAFLD and hyperglycemia due to T2DM.

**Variable (mean±SE)**	**Patients with NAFLD**	
	**Without T2DM [Group A] (n=58)**	**With T2DM [Group B] (n=47)**
Age (years)	53.8±4.9	52.7±5.1
Gender (m/f)	35/23	31/16
Systolic blood pressure (mm Hg)	122.4±11.2	126.3±9.7
Diastolicblood pressure (mm Hg)	84.6±4.1	89.4±4.6
Body mass index (Kg/m^2^)	27.8±2.0	29.4±1.8
Waist/hip ratio	0.92±0.04	0.93±0.07
ALT (U/l)	30.5±2.8	34.8±3.2
AST (U/l)	28.4±2.7	28.9±2.1
GGT (U/l)	24.4±1.7	27.3±2.1
Fasting glucose (mg/dl)	89.4±7.8	203.6±4.7*
Fasting insulin (mU/l)	17.3±1.4	19.8±1.7
HOMA IR	4.1±0.5	9.4±0.79*

NAFLD...non alcoholic fatty liver disease; T2DM...type 2 diabetes mellitus;

HOMA insulin resistance (using the formula of Mathews) = $\frac{\left(\text{fasting}\;\text{insulin}\times \text{fasting}\;\text{plasma}\;\text{glucose}\right)}{405}$

* Significant difference at p<0.05.

**Tab. 2.** Lipid profile, oxidative stress and proinflammatory cytokine levels in patients with NAFLD in comparison to the patients with NAFLD and hyperglycemia due to T2DM.

**Variable (mean±SE)**	**Patients with NAFLD**	
	**Without T2DM [Group A] (n=58)**	**With T2DM [Group B] (n=47)**
Cholesterol (mg/dl)	209.4±12.8	221.7±19.6
Triglycerides (mg/dl)	174.4±10.1	206.7±13.3*
LDL-C (mg/dl)	144.3±9.7	175.7±7.6*
HDL-C (mg/dl)	29.7±2.7	21.4±2.1*
Leukocyte hydrogen peroxide (μM)	81.4±6.2	102.2±3.1*
Serum MDA (nmol/ml/h)	294.7±11.4	425.5±9.6*
Serum TNF-α(pg/ml)	11.7±1.2	21.1±0.6*
Serum IL-6 (pg/ml)	28.2±2.8	40.4±1.0*

NAFLD...non alcoholic fatty liver disease; T2DM...type 2 diabetes mellitus; LDL...low density lipoprotein; HDL...high density lipoprotein; MDA...malondialdehyde; TNF-α...tumor necrosis factor-alpha; IL-6...interleukin-6.

* Significant difference at p<0.05.
